# Subtotal gastrectomy pancreaticoduodenectomy versus conventional pancreaticoduodenectomy in the incidence of delayed gastric emptying: single-center retrospective cohort study

**DOI:** 10.1186/s12893-022-01824-4

**Published:** 2022-11-04

**Authors:** Jinzhu Zhang, Shu Li, Weihua Zhu, Xisheng Leng, Jie Gao, Dafang Zhang

**Affiliations:** Department of Hepatobiliary Surgery, Peking University People’s Hospital, No. 11 Xizhimen South Street, Xicheng District, Beijing, 100044 China

**Keywords:** Pancreaticoduodenectomy, Subtotal gastrectomy, Surgery, Delayed gastric emptying, General surgery, Complication

## Abstract

**Background:**

Delayed gastric emptying (DGE) is one of the most common complications after pancreaticoduodenectomy (PD). There is currently no widely accepted procedure for PD to reduce the incidence of DGE. Our institution attempts to perform subtotal gastrectomy in patients undergoing PD to reduce DGE. Here we aimed to evaluate the effectiveness and safety of PD with subtotal gastric resection.

**Methods:**

Patients who underwent PD between January 2014 and December 2021 were reviewed. They were stratified by extent of gastrectomy into a conventional PD group (PD that resected approximately 1/3 of the distal stomach) and a subtotal gastrectomy PD group (PD that resected approximately 3/4 of the distal stomach), which were compared in terms of intraoperative and postoperative parameters.

**Result:**

From January 2014 to December 2021, a total of 512 patients underwent PD in the Department of Hepatobiliary Surgery, Peking University People’s Hospital. Nineteen patients were excluded from this study due to benign disease. A total of 493 patients were included, with 378 in the conventional PD group and 115 in the subtotal gastrectomy PD group. Compared with the conventional PD group, the subtotal gastrectomy PD group had a lower incidence of DGE (8.7% vs. 17.7%, p = 0.019), and a shorter hospital stay. Multivariate analysis showed that conventional PD and higher body mass index were independent risk factors for grade B/C DGE.

**Conclusion:**

This study showed that, compared with conventional PD, subtotal gastrectomy PD can reduce the incidence of DGE and shorten the length of hospital stay. At the same time, subtotal gastrectomy PD is comparable to conventional PD in terms of surgical safety. Furthermore, high BMI is an independent risk factor for postoperative DGE.

## Introduction

Pancreaticoduodenectomy (PD) is used for the treatment of pancreatic head cancer, cholangiocarcinoma, ampullary carcinoma and duodenal carcinoma [1]. Despite advances in surgical techniques for PD, there is a high rate of postoperative complications, including delayed gastric emptying (DGE), pancreatic fistula, hemorrhage, biliary fistula and abdominal infection [2]. DGE is one of the most common complications after PD, with an incidence of approximately 17.5–56.0% [3, 4]. DGE does not directly cause the death of patients. However, prolonged feeding difficulty after surgery increases the patients’ hospital stay and medical costs.

The mechanism of DGE is currently not fully understood. Researchers attempted to explain DGE from different perspectives, such as smooth muscle dysfunction, enteric nervous system damage, pyloric sphincter disorder, oxidative distress and inflammation [5].

Surgeons have been working to improve surgical techniques for PD to reduce the incidence of postoperative gastric emptying disorders. However, it is currently unclear which surgical approach can reduce the incidence of DGE. In the past, surgeons believed that the incidence of DGE was related to the method for digestive tract reconstruction and whether the pylorus was preserved [6–8]. Few studies have investigated the association between the extent of gastrectomy and the incidence of DGE after PD. Since 2014, our institution has performed subtotal gastrectomy in some patients undergoing PD. Here we aimed to evaluate the effectiveness and safety of PD with subtotal gastric resection.

## Materials and methods

### Population

Patients who underwent PD at the Department of Hepatobiliary Surgery, Peking University People’s Hospital from January 2015 to December 2021 were reviewed. Patients who underwent PD for benign disease were excluded. According to the extent of distal gastrectomy, the subjects were divided into a conventional PD group (PD that resected approximately 1/3 of the distal stomach) and a subtotal gastrectomy PD group (PD that resected approximately 3/4 of the distal stomach). Surgical notes in the medical record system describe the extent of distal gastrectomy in PD. The following parameters were included as possible confounders: age, sex, body mass index (BMI), American Society of Anesthesiologists (ASA) grade, preoperative comorbidities, and site of lesion. The following parameters were compared: operative time, blood loss, incidence of complications, hospital stay and number of in-hospital deaths.

### Surgery

**Fig. 1:**
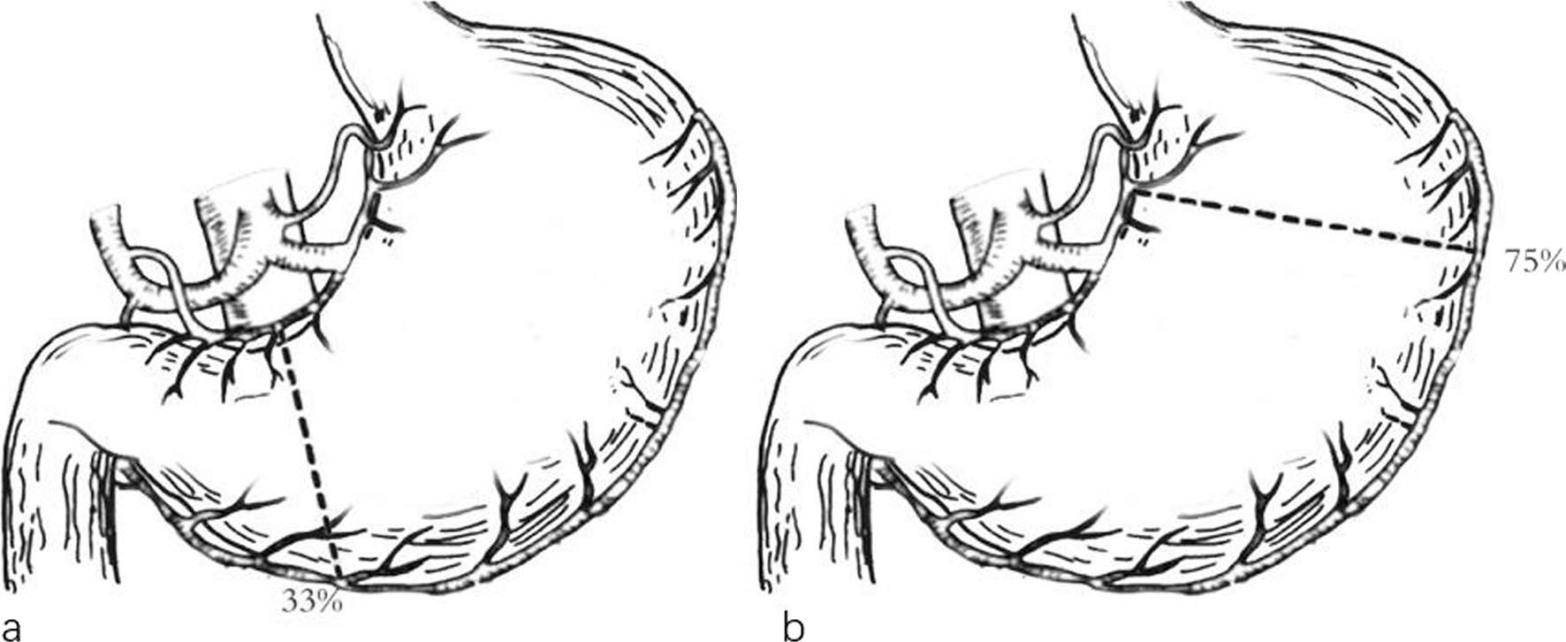
Illustration of the two PD. **a** Conventional PD removes approximately 1/3 of the distal end of the stomach. The resection line for the stomach is located just above the gastric antrum. **b** Subtotal gastrectomy PD removes the distal 3/4 of the stomach. The resection line crosses the second branch of the left gastric artery and the avascular area on the left side of the stomach

Except for the extent of gastrectomy, conventional PD and subtotal gastrectomy PD have the same surgical procedures. Roux-en-y or child surgery was used to reconstruct the digestive tract. Conventional PD surgery removes approximately 1/3 of the distal end of the stomach. The resection line for the stomach is located just above the gastric antrum. Subtotal gastrectomy PD removes the distal 3/4 of the stomach. The resection line crosses the second branch of the left gastric artery and the avascular area on the left side of the stomach (Fig. 1)

### Definition of DGE

According to the definition by the International Study Group of Pancreatic Surgery [9], grade A DGE was defined as a patient requiring retention of the nasogastric tube for 4–7 days after surgery, 8–14 days for grade B DGE, and more than 14 days for grade C DGE.

### Postoperative management

After surgery, when the drainage volume of gastrointestinal decompression was less than 150 ml per day, the nasogastric tube was removed. Abdominal drainage fluid amylase was regularly monitored daily for 5 days after surgery. After the patient’s gastrointestinal tract recovered peristalsis, liquid food would be admitted. Semi-liquid and solid foods would be gradually allowed according to patient tolerance.

### Statistical analysis

Data were analyzed in Statistical Package for the Social Sciences version 21.0. When a continuous variable conformed to a normal distribution, the variable was expressed as mean ± standard deviation. Otherwise, the variable was expressed with the median and interquartile range. Continuous variables between groups were compared by Student’s t-test and Mann–Whitney U test according to whether they were normally distributed. Categorical variables were analyzed by chi-square test. Univariate and multivariable logistic regression was used to assess the risk factor on outcome. Variables with p values less than 0.05 were included in the multivariable logistic regression analysis. P values less than 0.05 were considered significant.

## Results

### Baseline characteristics

From January 2015 to December 2019, a total of 512 patients underwent PD at the Department of Hepatobiliary Surgery, Peking University People’s Hospital. Nineteen patients were excluded from the study due to postoperative pathology confirmed to be benign. A total of 493 patients were included in this study.

The baseline information of the patients is shown in Table 1. Of the 493 patients, 378 patients underwent conventional PD with distal 1/3 gastric resection, and 115 patients underwent subtotal gastrectomy PD with distal 2/3 gastric resection. There were no significant differences in any baseline information between the conventional PD group and the subtotal gastrectomy PD group (Table 1).

**Table 1:** Baseline characteristics

	Control PD group (n = 378)	Subtotal gastrectomy PD group (n = 115)	P value
Age (yr)	64 (57–69)	64.0 ± 10.0	0.631
Gender, n (%)			1.000
Male	236 (62.4)	72 (62.6)	
Female	142 (37.6)	43 (37.4)	
Jaundice, n (%)	214 (56.6)	60 (52.2)	0.453
Diabetes, n (%)	99 (26.2)	26 (22.6)	0.465
Hypertension, n (%)	122 (32.3)	34 (29.6)	0.647
Coronary atherosclerotic heart disease, n (%)	23 (6.1)	4 (3.5)	0.355
History of Abdominal Surgery, n (%)	46 (12.2)	17 (14.8)	0.523
BMI (kg/m^2^)	23.0 (20.8–25.3)	22.9 ± 3.4	0.503
ALT/AST Elevation, n (%)	255 (67.5)	69 (60.0)	0.146
ASA classification, n (%)			0.474
I	26 (6.9)	6 (5.2)	
II	296 (78.3)	87 (75.7)	
III	56 (14.8)	22 (19.1)	
Site of lesion, n (%)			0.134
Pancreas	141 (37.3)	53 (46.1)	
Bile duct	142 (37.6)	20 (17.4)	
Duodenum	95 (25.1)	42 (36.5)	

### Intraoperative and postoperative parameters

The intraoperative and postoperative parameters are shown in Table 2. Compared with the conventional PD group, the subtotal gastrectomy PD group had a lower incidence of grade B/C DGE and a shorter hospital stay. There were no differences in other parameters (Table 2). No patient died intraoperatively. Nineteen patients (3.7%) died during postoperative hospitalization. Four patients died of pneumonia; four patients died of abdominal infection; and five patients died of gastrointestinal bleeding or intra-abdominal bleeding. Four patients died of pancreatic fistula. One patient died of biliary fistula; one person died of thromboembolism.

**Table 2:** Intraoperative and postoperative parameters

	Control PD group (n = 378)	Subtotal gastrectomy PD group (n = 115)	P value
Operative time (min)	420 (360–502)	410 (369.5–463)	0.336
Technique of reconstruction			0.195
Roux-en-Y	197 (52.1)	52 (45.2)	
Child surgery	181 (47.9)	63 (54.8)	
Blood loss (ml)	600 (400–950)	600 (400–900)	0.637
Postoperative pancreatic fistula (B/C), n (%)	53 (14.0)	18 (15.7)	0.651
DGE (B/C), n (%)	67 (17.7)	10 (8.7)	0.019
Biliary fistula, n (%)	12 (3.2)	2 (1.7)	0.537
Gastrointestinal bleeding, n (%)	28 (7.4)	5 (4.3)	0.294
Intra-abdominal bleeding, n (%)	20 (5.3)	6 (5.2)	1.000
Abdominal infection, n (%)	28 (7.4)	12 (10.4)	0.329
Postoperative hospital stay (d)	18 (13–30)	15 (12–23)	0.003
In-hospital death, n (%)	13 (3.4)	6 (5.2)	0.408

### Univariate and multivariate logistic regression analysis of the risk factors for DGE

To identify the risk factors for DGE, we performed univariate and multivariate logistic regression analyses. The univariate analysis identified conventional PD and high BMI as risk factors for grade B or C DGE. In the multivariate logistic regression, conventional PD and high BMI were independent risk factors for grade B/C DGE (Table 3).

**Table 3:** univariate and multivariate analyses of risk factors for delayed DGE (B/C)

Characteristics	Univariate analysis			Multivariate analysis		
	P value	Odds ratio	95% CI	P value	Odds ratio	95% CI
Age (yr) > 60	0.913	1.029	0.621–1.705			
Male	0.211	1.394	0.828–2.348			
Jaundice, n (%)	0.195	1.392	0.844–2.294			
Diabetes, n (%)	0.316	0.739	0.409–1.336			
Hypertension, n (%)	0.663	1.122	0.670–1.879			
Coronary atherosclerotic heart disease, n (%)	0.670	1.244	0.456–3.391			
History of Abdominal Surgery, n (%)	0.755	0.887	0.418–1.882			
BMI (kg/m2) > 23	0.017	1.828	1.112–3.004	0.022	1.792	1.087–2.953
ALT/AST Elevation, n (%)	0.715	1.101	0.656–1.849			
ASA classification, n(%)						
I	Reference					
II	0.502	0.727	0.286–1.846			
III	0.834	1.118	0.394–3.117			
Site of lesion, n (%)						
Pancrea	Reference					
Bile duct	0.521	1.189	0.701–2.017			
Duodenum	0.057	0.482	0.227–1.022			
Technique of reconstruction						
Roux-en-Y	Reference					
Child surgery	0.474	1.195	0.734–1.945			
Operative time (min) > 400	0.677	1.110	0.679–1.814			
Blood Loss (ml) > 600	0.972	0.991	0.608–1.615			
Subtotal gastrectomy PD, n (%)	0.022	0.442	0.219–0.890	0.027	0.453	0.224–0.915

## Discussion

Previous study reported that subtotal gastrectomy has a lower incidence of DGE of only 3.1% [10]. Thus, we hypothesized that a less remnant stomach might lead to a lower incidence of DGE and attempted subtotal gastrectomy for PD. Conventional PD surgery requires removal of approximately 1/3 of the distal stomach. Compared with conventional PD, subtotal gastrectomy PD removes approximately 3/4 of the stomach. Several investigators have reported that the subtotal gastrectomy in PD surgery may reduce the incidence of postoperative DGE. Yusuke et al. compared the incidence of DGE between subtotal stomach-preserving PD and antrectomy-combined PD and finally concluded that antrectomy-combined PD leads to a lower incidence of DGE. Philip et al. [11] reported that 4/5 gastrectomy in patients undergoing PD reduces the incidence of DGE. Toshihiko et al. [12]. reported that Roux-en-Y reconstruction following gastric cancer was more frequently followed by Roux stasis in the antrum than in the midstomach.

Our study compared the short-term outcomes of subtotal gastrectomy PD and conventional PD. Compared with conventional PD, subtotal gastrectomy PD resulted in a lower incidence of DGE B/C (17.7% vs. 8.7%) and a shorter hospital stay. This result is similar to previous studies. Due to the lower incidence of DGE, subtotal gastrectomy PD had a shorter hospital stay. Subtotal gastrectomy PD was similar to conventional PD in terms of intraoperative bleeding, operative time and in-hospital mortality. This means that although PD removed more stomach, it did not increase the morbidity and mortality of patients.

Currently, DGE is thought to be mainly caused by pyloric dysfunction and impairment of the propulsive action of the stomach [13]. Some publications have reported that pylorus-resecting PD results in a lower incidence of DGE than pylorus-preserving PD [6, 14]. The elimination of pyloric dysfunction caused by pyloric resection may be the reason for the lower incidence of DGE in pyloric-resecting PD. Additionally, the smaller remnant stomach volume increases the mechanical stimulation of the stomach by food, which can promote the vago-vagal reflex and the local reflex of the intramural plexus, thereby enhancing gastric peristalsis and promoting gastric emptying [15].

Our study did not analyze the long-term quality of life of patients after PD. Yusuke et al. compared the nutritional status after PD between the antrectomy-combined PD group and the subtotal stomach-preserving PD group. The nutritional status of the two groups at 3, 6, and 12 months after surgery was comparable. Santoro et al. reported that most patients who underwent subtotal gastrectomy showed no significant difference in long-term quality of life after surgery compared with preoperative patients [16]. Philip et al. reported that subtotal gastrectomy PD patients with subtotal gastrectomy lost more body weight 1.5 months after surgery than conventional PD patients. However, there was no significant difference in weight loss between the two groups at 3 months and 6 months after surgery. Additionally, most of the patients who received PD in our center did not complain of obvious discomfort in the postoperative outpatient follow-up. Therefore, our center initially believes that subtotal gastrectomy PD has little adverse effect on the long-term quality of life of patients.

In the multivariate regression analysis, conventional PD and elevated BMI were risk factors for DGE. Previous studies have also confirmed that high BMI is associated with an increased risk of DGE [17, 18]. A high BMI can lead to the accumulation of fat around the patient’s organs, which increases the difficulty of PD surgery.

In addition to conventional PD and subtotal gastrectomy PD, pylorus-preserving PD is the standard of care in many institutions. Many studies have reported that pylorus-preserving PD can reduce operating time, intraoperative blood loss and postoperative hospital stay compared with conventional PD [19, 20]. However, some studies reported that pylorus-preserving PD increased the incidence of postoperative complications, DGE^21^, ^22^]. Which surgical approach maximizes patient benefit may require further research.

This study preliminarily confirmed that subtotal gastric resection of PD can effectively reduce the incidence of postoperative DGE, and the level of safety is similar to that of conventional PD. However, the study still has some limitations, as it was a single-center, retrospective cohort study. Further multicenter prospective studies are needed in the future. Additionally, further long-term quality of life assessments should be conducted in future investigations.

## Conclusion

This study showed that, compared with conventional PD, subtotal gastrectomy PD can reduce the incidence of DGE and shorten the length of hospital stay. At the same time, subtotal gastrectomy PD is comparable to conventional PD in terms of surgical safety. Furthermore, high BMI is an independent risk factor for postoperative DGE. The long-term quality of life in patients who undergo subtotal gastrectomy PD requires further study.

## Data Availability

The datasets analyzed during the current study are available from the corresponding author on reasonable request.

## Abbreviations

PD: Pancreaticoduodenectomy
DGE: Delayed gastric emptying
BMI: Body mass index
ASA: American Society of Anesthesiologists
